# Tuberculosis in the Russian Federation: Prognosis and Epidemiological Models in a Situation After the COVID-19 Pandemic

**DOI:** 10.1007/s44197-023-00085-5

**Published:** 2023-02-06

**Authors:** Anna Starshinova, Ekaterina Belyaeva, Natalia Doktorova, Ilya Korotkevich, Dmitry Kudlay

**Affiliations:** 1 Almazov National Medical Research Centre, 197341 St. Petersburg, Russia; 2 The Republic of Karelia “The Republic TB Healthcare Dispensary”, 185032, Petrozavodsk, Russia; 3 GSC «GENERIUM», 123112 Moscow, Russia; 4 First Moscow State Medical University (Sechenov University), 119991, Moscow, Russia; 5 NRC Institute of Immunology FMBA of Russia, 115522, Moscow, Russia

**Keywords:** Tuberculosis, COVID-19, Epidemiological models, Screening, Incidence, Mortality

## Abstract

**Aim:**

Because of the COVID-19 pandemic, many support programs for tuberculosis (TB) patients have been discontinued and TB mass screening activities decreased worldwide, resulting in a decrease in new case detection and an increase in TB deaths (WHO, WHO global lists of high burden countries for TB, multidrug/rifampicin-resistant TB (MDR/RR-TB) and TB/HIV, 2021–2025, 2021). The study aimed to assess changes in epidemiological indicators of tuberculosis in the Russian Federation and to simulate these indicators in the post-COVID-19 period.

**Materials and Methods:**

The main epidemiological indicators of tuberculosis were analyzed with the use of government statistical data for the period from 2009 to 2021. Further mathematical modeling of epidemiological indicators for the coming years was carried out, taking into account the TB screening by chest X-ray. Statistical analysis was carried out using the software environment R (v.3.5.1) for statistical computing and the commercial software Statistical Package for the Social Sciences (SPSS Statistics for Windows, version 24.0, IBM Corp., 2016). Time series forecasting was performed using the programming language for statistical calculations R, version 4.1.2 and the bsts package, version 0.9.8.

**Study Results:**

The study has found that the mean regression coefficient of a single predictor differs in the model for TB incidence and mortality (0.0098 and 0.0002, respectively). Forecast of overall incidence, the incidence of children and the forecast for mortality using the basic scenario (screening 75–78%) for the period from 2022 to 2026 was characterized by a mean decrease rate of 23.1%, 15.6% and 6.0% per year, respectively. A conservative scenario (screening 47–63%) of overall incidence indicates that the incidence of children and the forecast for mortality will continue to decrease with a mean decrease rate of 23.2%, 15.6% and 6.0% per year, respectively. Comparable data were obtained from the forecast of overall incidence, the incidence of children and the forecast for mortality using the optimistic scenario (screening 82–89%) with a mean decrease rate of 22.9%, 15.4% and 6.0% per year, respectively.

**Conclusions:**

It has been proven that the significance of screening with chest X-ray as a predictor of mortality is minimal. However, TB screening at least 60% of the population (chest X-ray in adults and immunological tests in children) have provided relationship between the TB screening rate and TB mortality rate (TB mortality rate increases with an increase in the population coverage and, conversely, decreases with a decrease in the population coverage).

## Introduction

At the beginning of the twenty-first century, tuberculosis remains a pressing issue that receives attention at various levels. Being one of the leading causes of death worldwide, it is still a marker of the country’s social and economic well-being [1–3].

Despite the introduction of various international projects and programs aimed at reducing the incidence of tuberculosis in the world (the Directly observed treatment, short-course (DOTS) program, the END TB Strategy and the Global Plan to End TB for 2006–2015), currently we cannot say they are sufficiently efficient and successful in the fight against the prevalence of tuberculosis [4].

In 2014, WHO introduced a new END TB strategy and global goals were set for the entire community: to reduce the absolute number of TB-related deaths by 90% and to reduce TB incidence by 80% (new cases per 100,000 people per year) by 2035, which will mean the eradication of the disease worldwide [5, 6]. More than 85 countries of the world, including the Russian Federation, endorsed this strategy.

The pandemic of a new coronavirus infection (COVID-19) has introduced significant adjustments to the TB care programs around the globe, which has affected the detection of new cases of TB in all countries of the world and the mortality due to the infection [7, 8].

The epidemiological models carried out in the study allowed predicting a 10% increase in TB-related mortality and a 20% increase in HIV-related mortality a year before receiving official statistics, which is due to a decrease in activities under existing prevention and diagnosis programs [9].

According to the latest WHO data, the COVID-19 pandemic has seriously undermined the progress made in the fight against this disease worldwide: TB-related mortality rates have reached 2015 levels for the first time in more than a decade [1, 10]. In 2020, compared to 2019, the number of people screened for tuberculosis significantly decreased, as well as decreased the scope of screening for latent TB infection, and funding for the main types of anti-tuberculosis care was drastically reduced, including due to repurposing of hospitals to provide care to COVID-19 patients [11, 12].

According to WHO prognosis, in 2021 and 2022, the number of people who get TB infection and die of it will be much higher [13]. According to WHO estimates, about 4.1 million TB patients have not yet been diagnosed or are not included in the official statistics of countries [8]. At the same time, the number of patients receiving treatment for drug-resistant tuberculosis decreased by 15%, from 177,000 people in 2019 to 150,000 people in 2020 [14, 15].

TB morbidity the pediatric population is a marker of the epidemiological situation in the country, since children usually become infected by *M. tuberculosis* into close contact with parents. Work in a focus of tuberculosis infection always reflects the efficiency and quality of the organization of activities aimed at detection of TB patients, especially exposed persons [16].

An analysis of the main epidemiological indicators for TB (overall incidence, incidence in children aged 0–17 years and mortality) suggests that the pandemic of the new coronavirus infection has led to a certain slowdown in improvement of these indicators, which may be related to the fact that chest X-ray examinations to detect active tuberculosis have not been carried in a sufficient number of persons. This situation may lead to an increase in the disease-related mortality and the incidence and severity of tuberculosis in children due to the growth of a core of smear-positive TB patients in the Russian Federation regions.

To understand the consequences of the COVID-19 pandemic, it is necessary to understand dynamic of TB epidemiology data. During the COVID-19 pandemic, no adequate screening examination of the population for tuberculosis was carried out, which is expected to negatively affect the epidemiological situation in the country and lead to an increase in the TB-related incidence and mortality in the population. The aim of the study was to assess changes in the epidemiological indicators of tuberculosis infection and mathematical modeling of these indicators in the Russian Federation after the COVID-19 pandemic in the coming years.

## Materials and Methods

An analysis of the main epidemiological indicators of tuberculosis using the state statistics (TB incidence rates, overall incidence in the population, incidence in children aged 0–17 years, etc.) was carried out, and the population coverage with chest X-ray examinations was assessed for the period from 2009 to 2021. Annual indicators were estimated per 100 thousand of the mid-year pediatric and adult population using the state statistics of the Russian Federation [17].

Time series forecasting was performed using the programming language for statistical calculations R, version 4.1.2 [18] and the bsts package, version 0.9.8 [19]. This package is based on the methodology of Bayesian structural time series models, where the time series was considered as the sum of various components: trend, seasonality and predictor effects.

Models using the following components were considered as candidate models for predicting the time series of overall TB incidence, TB incidence in children (aged 0–17 years) and TB mortality:

- only with a trend (local linear or semi-local linear);
- with a trend and autoregression;
- with a trend, autoregression and a predictor—the population coverage with screening examinations for tuberculosis using chest X-ray.

The local linear trend is described by the mean level of a time series and its increase/decrease rate. The semi-local linear trend component differs from the normal linear trend, in that an increase/decrease rate in the mean level of a series is regulated by first-order autoregression (with a lag of 1) [20].

In models with predictors, their regression coefficients were also subjected to a regularization process: it was initially assumed that the inclusion probability of a predictor in the model was zero, and the inclusion probability was updated as the algorithm ran. As a result, it showed the probability of using the predictor in individual model implementations, including forecasting.

One-step-ahead errors were calculated during the fitting of each model using the training data, which were complete time series of the indicated epidemiological indicators.

The sample for determining forecast errors on test data was not selected, as time series analyzed were too short (2009–2021, 13 years). The dynamics of epidemiological indicators for 2020–2021 differed from that in previous periods because of the lack of adequate testing for tuberculosis due to restrictions during the COVID-19 pandemic. In these years, the dependence of epidemiological indicators on the coverage with screening examinations was the opposite to the expected one (the values of the indicators decreased with a decrease in coverage and increased with an increase in coverage). The indicators of this time period were not used as test data.

Increase/decrease rates were used for a descriptive analysis of the used time series of epidemiological indicators. They were calculated using to the following formula:

$$\frac{\mathrm{Increase}}{\mathrm{decrease\,rate}}\,=\,{(X}_{\mathrm{c}}/{X}_{\mathrm{pr}}\times 100\%)-100\%,$$

where *X*_c_—the value of the indicator at the current moment, and *X*_pr_—the value of the indicator at the previous time point.

Unless otherwise specified, the increase/decrease rate relative to the previous year was used in all cases. To describe the levels of incidence and mortality in children in 2021, the decrease rate relative to 2019 was used.

Harvey’s goodness of fit statistic [20], which depends on one-step-ahead errors and the number of observations in the analyzed time series, was used as a metric for a comparative assessment of the quality of the models.

Time series forecasting of the overall tuberculosis incidence, the tuberculosis incidence in children (0–17 years of age) and TB-related mortality was carried out for a 5-year period. If the addition of a predictor to the model improved it and the probability of its inclusion in the model was non-zero, the effect of the predictor on the target indicator was considered proven. In this case, the forecasting of the target indicator values for the next 5 years was performed according to the following three scenarios:

- basic scenario—the current dynamics of the coverage with screening examinations for tuberculosis remaining unchanged;
- conservative scenario—a sharp decrease in the coverage with screening examinations for tuberculosis;
- optimistic scenario—a sharp increase in the coverage with screening examinations for tuberculosis.

For the basic scenario, we used a forecast of the screening examination (SE) coverage over a 5-year period, obtained on a time series model using a linear trend and autoregression with a maximum lag of 3. The number of algorithm iterations was 10,000, and the value of Harvey’s goodness-of-fit statistics was 0.1121245 (≈0.11).

Predicted scenarios using expert medical assessments of the actual coverage with screening examinations for tuberculosis are presented in Table 1.

**Table 1:** Predictive values of the coverage with TB screening examinations

Model scenario	Years and population coverage with screening examinations for tuberculosis (%)				
	2022	2023	2024	2025	2026
Basic (current trend unchanged)	75.89	76.74	77.04	77.66	78.15
Conservative (sharp decrease)	47.37	58.95	60.00	62.11	63.16
Optimistic (sharp increase)	82.11	85.26	86.32	88.42	89.47

### Study Results

Mathematical modeling of the overall incidence of tuberculosis in the Russian Federation is built using the following components:

- linear trend (Model 1);
- linear trend and autoregression with a maximum lag of up to 5 (Model 2);
- linear trend and autoregression with a maximum lag of up to 5 and predictor (Model 3).

The values of Harvey’s goodness-of-fit statistics were:

- 0.05239519 (≈0.05) for Model 1,
- 0.06460138 (≈0.06) for Model 2,
- 0.07917504 (≈0.08) for Model 3.

According to the data obtained, Model 3 with components in the form of a linear trend, autoregression, and the population coverage with SE for TB as a predictor is optimal. The probability of including this predictor in individual model implementations is 41.3%. Further, on the basis of this model, forecasts of the overall incidence of tuberculosis in the population of the Russian Federation were made.

It should be noted that if the value of the same statistics is positive, but close to zero, the benefit of a more complex model can be considered insignificant. Adding a predictor to Model 3 with linear trend and autoregressive components improved its predictive ability, but with low efficiency.

The mean value for the regression coefficient of a single predictor in Model 3 is 0.0098, which is very low. The value approaches zero, which indicates a very small contribution of the predictor to Model 3.

At the same time, the positive regression coefficient suggests a direct relationship between the SE coverage and the overall incidence of tuberculosis (the overall incidence increases with an increase in coverage and, conversely, decreases with a decrease in coverage). The linear trend and autoregression components contribute to Model 3 the most.

The results of predicting the overall tuberculosis incidence in the Russian Federation according to Model 3 are presented in Table 2.

**Table 2:** Forecast of the overall tuberculosis incidence of in the Russian Federation according to the created scenarios for a 5-year period

Prognosis	Years				
	2022	2023	2024	2025	2026
Conservative SE coverage (sharp decrease), %	47.37	58.95	60	62.11	63.16
Forecast according to the conservative scenario, per 100,000 population	26.06	21.92	17.48	13.27	8.96
Basic SE coverage (current trend unchanged), %	75.89	76.74	77.04	77.66	78.15
Forecast according to the basic scenario, per 100,000 population	26.31	22.07	17.63	13.4	9.08
Optimistic SE coverage (sharp increase), %	82.11	85.26	86.32	88.42	89.47
Forecast according to the optimistic scenario, per 100,000 population	26.36	22.15	17.71	13.5	9.18

Below is a forecast of the overall incidence of tuberculosis in the Russian Federation for each of the developed scenarios.

**Fig. 1:**
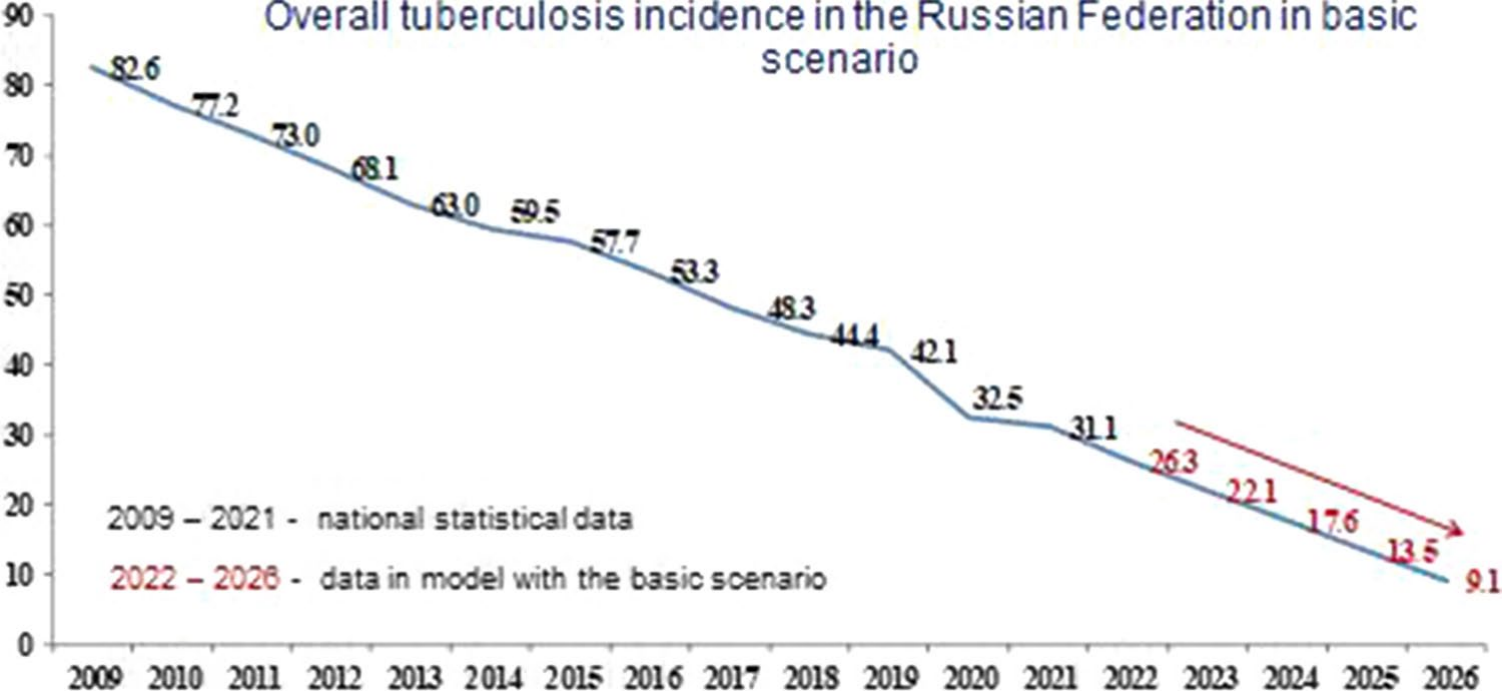
Forecast of the overall tuberculosis incidence in the Russian Federation with the basic scenario

**Fig. 2:**
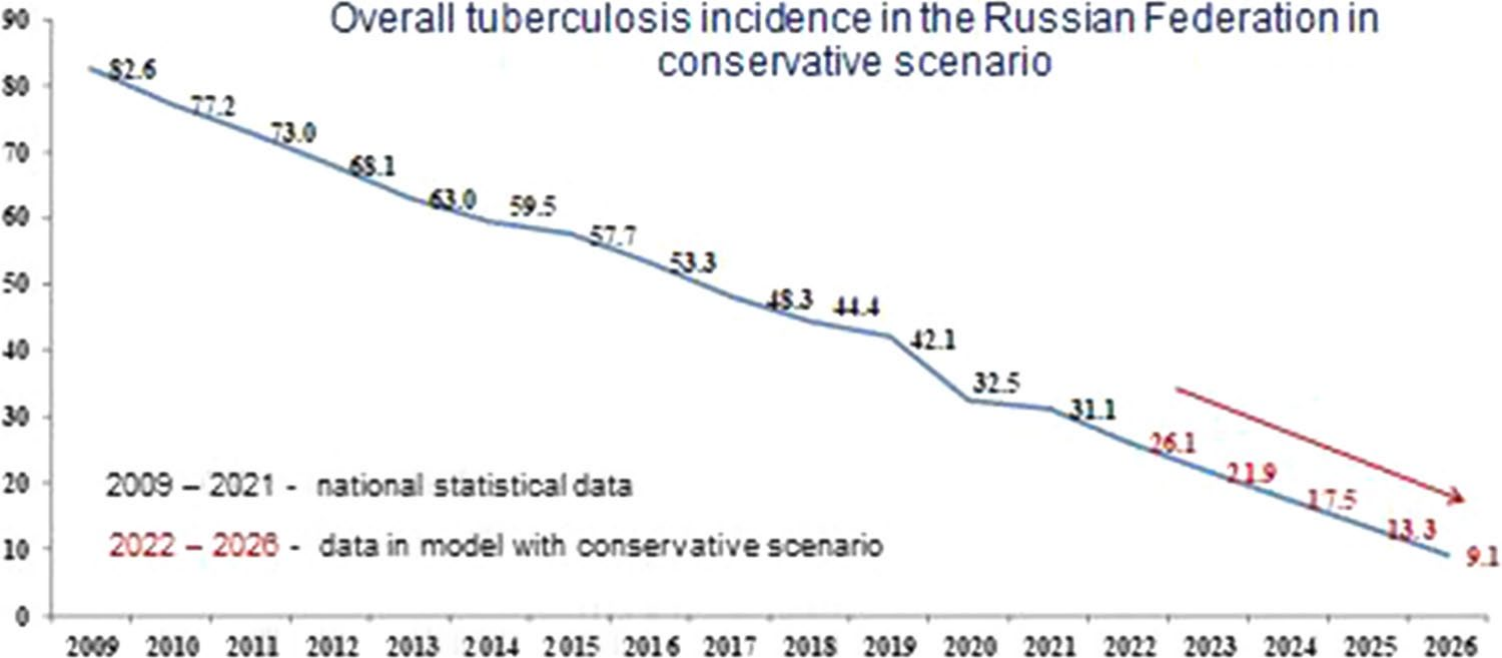
Forecast of the overall tuberculosis incidence in the Russian Federation with the conservative scenario

**Fig. 3:**
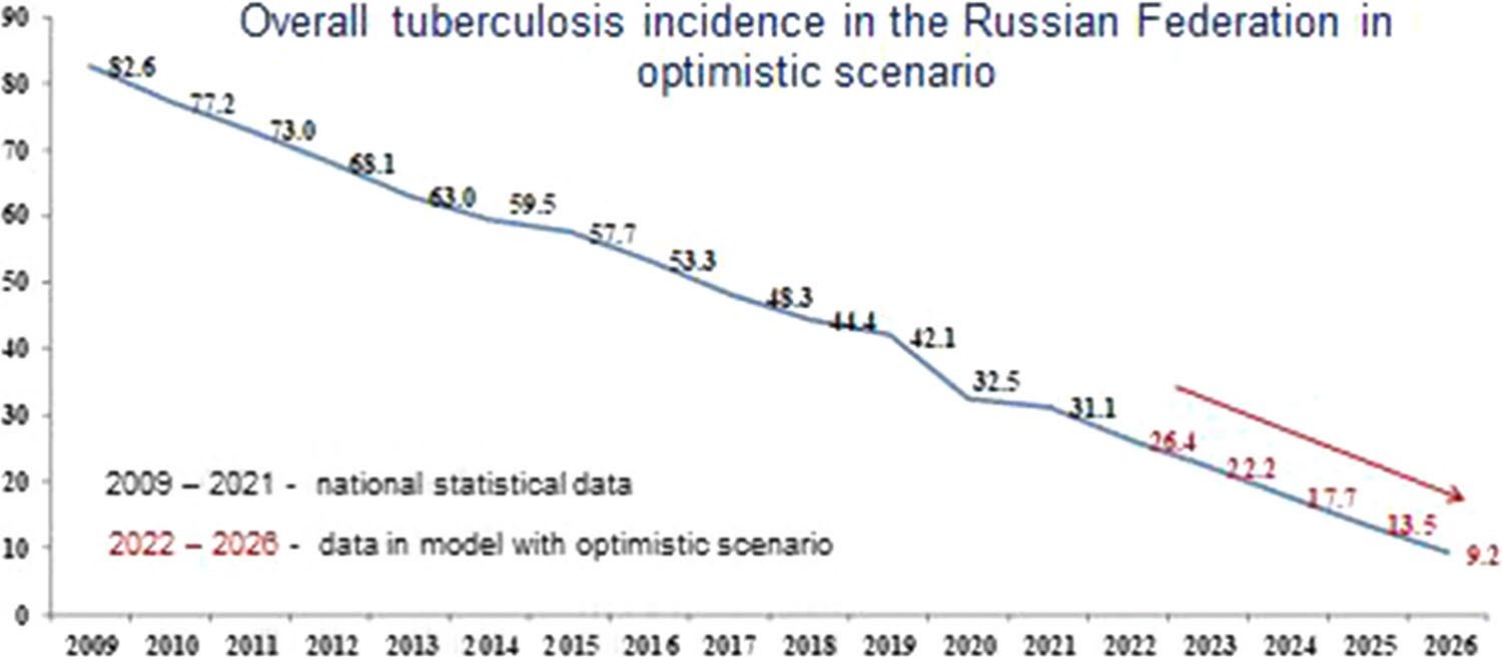
Forecast of the overall tuberculosis incidence in the Russian Federation with the optimistic scenario

Figure 1 shows the forecast for the basic scenario. Figures 2 and 3 show the results of the overall incidence of tuberculosis in the Russian Federation with the conservative and optimistic scenarios, respectively. According to the data presented in Figs. 1, 2 and 3, regardless of the scenario, there is a systematic decrease in the overall incidence of tuberculosis, regardless of the population coverage with SE for tuberculosis. Forecast of overall incidence using the basic scenario for the dynamics of SE coverage for the period from 2022 to 2026 is characterized by a persistent downward trend with a decrease rate in the range of 16.1–32.2% and a mean decrease rate of 23.1% per year (Fig. 1). Using a conservative scenario for the dynamics of SE coverage, the overall incidence will continue to decrease with a decrease rate in the range of 15.9–32.5% and a mean decrease rate of 23.2% per year (Fig. 2). Comparable data were obtained from the forecast of overall incidence using the optimistic scenario, where a decrease rate was observed within the range of 16.0–32.0% and a mean decrease rate of 22.9% per year (Fig. 3).

### Tuberculosis Incidence in Children Aged 0–17 Years

Models for the time series of TB incidence in children (0–17 years old) in the Russian Federation were built using the following components: local linear trend, semi-local linear trend, autoregression, with the addition of a predictor, as well as various combinations of them. Most of the models were rejected due to the negative values of Harvey’s goodness-of-fit statistics.

The analysis allowed identifying only two scenario models that showed minimal accuracy:

- with a semi-local linear trend (Model 1);
- with semi-local linear trend and predictor (Model 2).

The values of the Harvey’s goodness-of-fit statistics for these models were:

- 0.07766001 (≈0.078) for Model 1;
- 0.07901242 (≈0.079) for Model 2.

The data obtained show that Model 2 is optimal. Adding a predictor to Model 2 slightly increased the accuracy of the model. The probability of including this predictor in individual model implementations was 35.1%. Model 2 was further used to make forecasts of TB incidence in children.

The mean value for the predictor regression coefficient for Model 2 is 0.0036. A positive regression coefficient indicates the presence of a direct relationship between the SE coverage and TB incidence in children (the incidence in children increases with an increase in coverage and, conversely, decreases with a decrease in coverage).

**Fig. 4:**
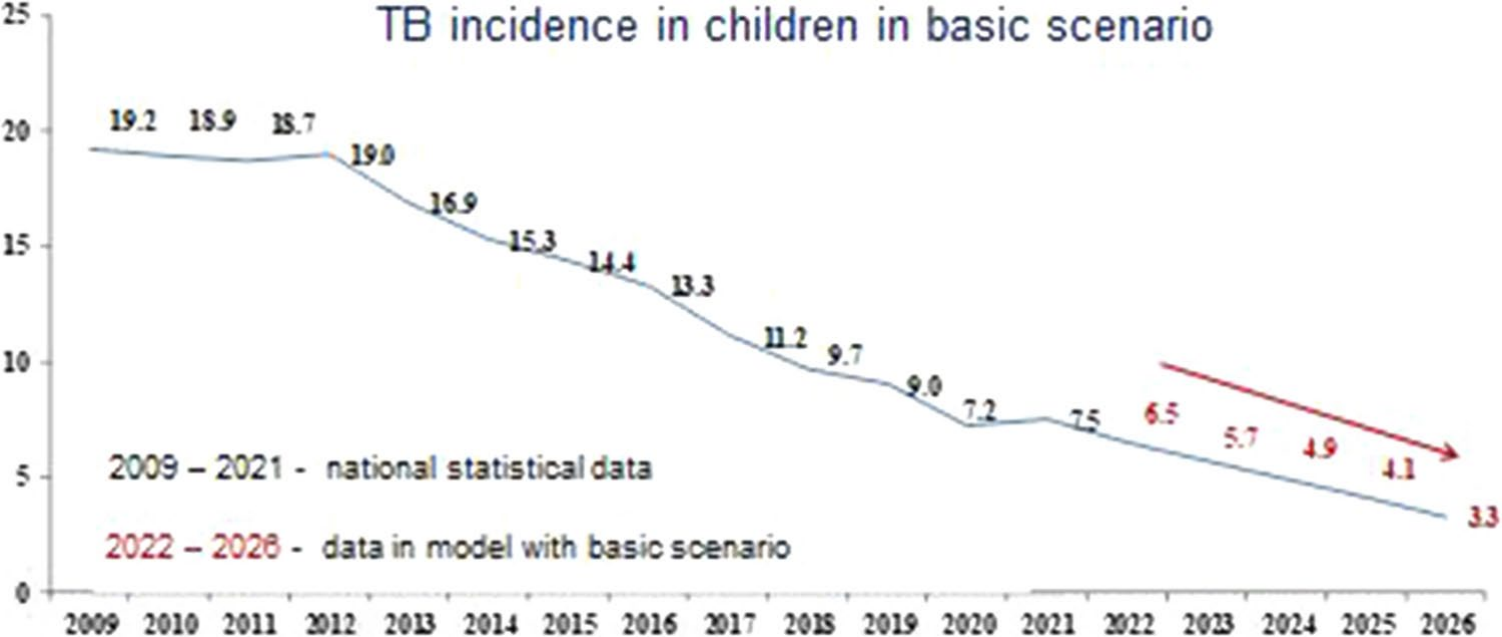
Forecast of the tuberculosis incidence in children (0–17 years) in the Russian Federation with the basic scenario

**Fig. 5:**
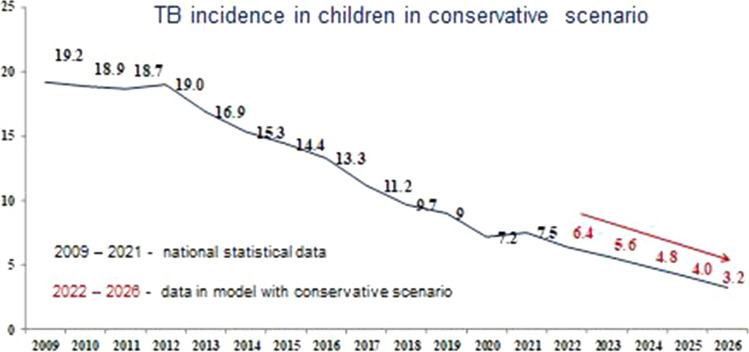
Forecast of the tuberculosis incidence in children (0–17 years) in the Russian Federation with the conservative scenario

**Fig. 6:**
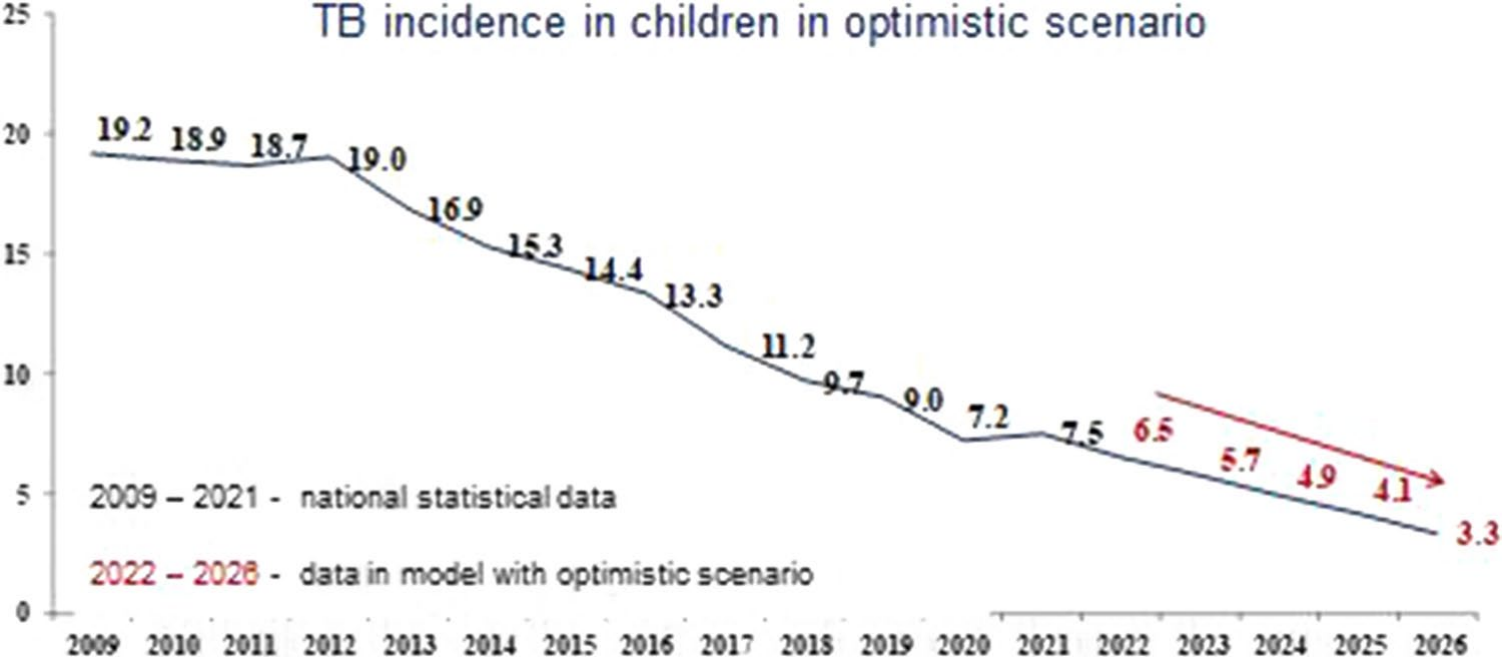
Forecast of the tuberculosis incidence in children (0–17 years) in the Russian Federation with the optimistic scenario

The results of forecasting tuberculosis incidence in children using the basic, conservative and optimistic scenarios are shown in Figs. 4, 5 and 6, respectively.

According to the data presented in the figures, the model predicts a decrease in the indicator with all scenarios of TB incidence in children, as well as for the overall TB incidence, regardless of the SE coverage.

With the basic scenario the dynamic of SE coverage, the forecast for the incidence of TB in children in the period from 2022 to 2026 is characterized by a confident downward trend with a decline rate in the range of 11.4–20.4% and a mean decrease rate of 15.6% per year (Fig. 4). Using a conservative scenario for the dynamics of SE coverage, this indicator will continue to decrease with a decrease rate in the range of 11.1–20.6% and a mean decrease rate of 15.6% per year (Fig. 5). Comparable data were obtained with the optimistic scenario of population SE coverage, where the incidence of TB in children would also continue to decrease with a decrease rate of 11.4–20.0% and a mean decrease rate of 15.4% per year (Fig. 6).

### TB-Related Mortality in the Russian Federation

Similar to the two previous indicators, models for the time series of TB-related mortality in the Russian Federation were built using the following components: local linear trend, semi-local linear trend, autoregression, with the addition of a predictor, as well as various combinations of them. Most of the models were also rejected due to the negative values of the Harvey’s goodness-of-fit statistics, which allowed conducting the analysis using Model 1 and Model 2 with a minimal accuracy.

The values of the Harvey’s goodness-of-fit statistics for these models were:

- 0.1740779 (≈0.17) for Model 1;
- 0.2009911 (≈0.20) for Model 2.

Like in the previous version, Model 2 was optimal, and the addition of a predictor in Model 1 made it possible to increase its accuracy. The probability of including this predictor in individual Model 1 implementations was 39.4%. This model was used to build further forecasts for TB-related mortality.

The mean regression coefficient of the predictor in this model was 0.0002, which indicates a low contribution of the predictor to the model. The positive regression coefficient indicates the presence of a direct relationship between the TB SE coverage and mortality from the disease (mortality increases with an increase in coverage and, conversely, decreases with a decrease in SE coverage).

Thus, the component of the local linear trend contributes the most to Model 1.

**Fig. 7:**
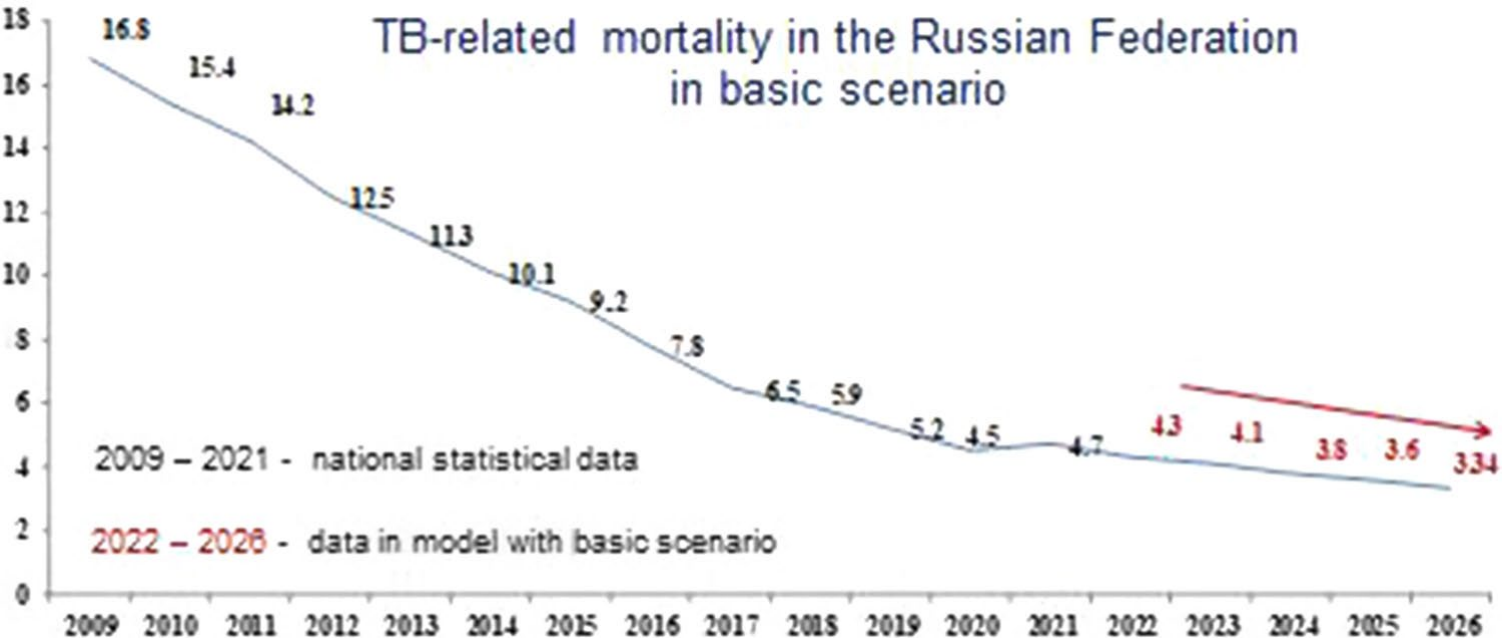
Forecast of TB-related mortality in the Russian Federation with the basic scenario

**Fig. 8:**
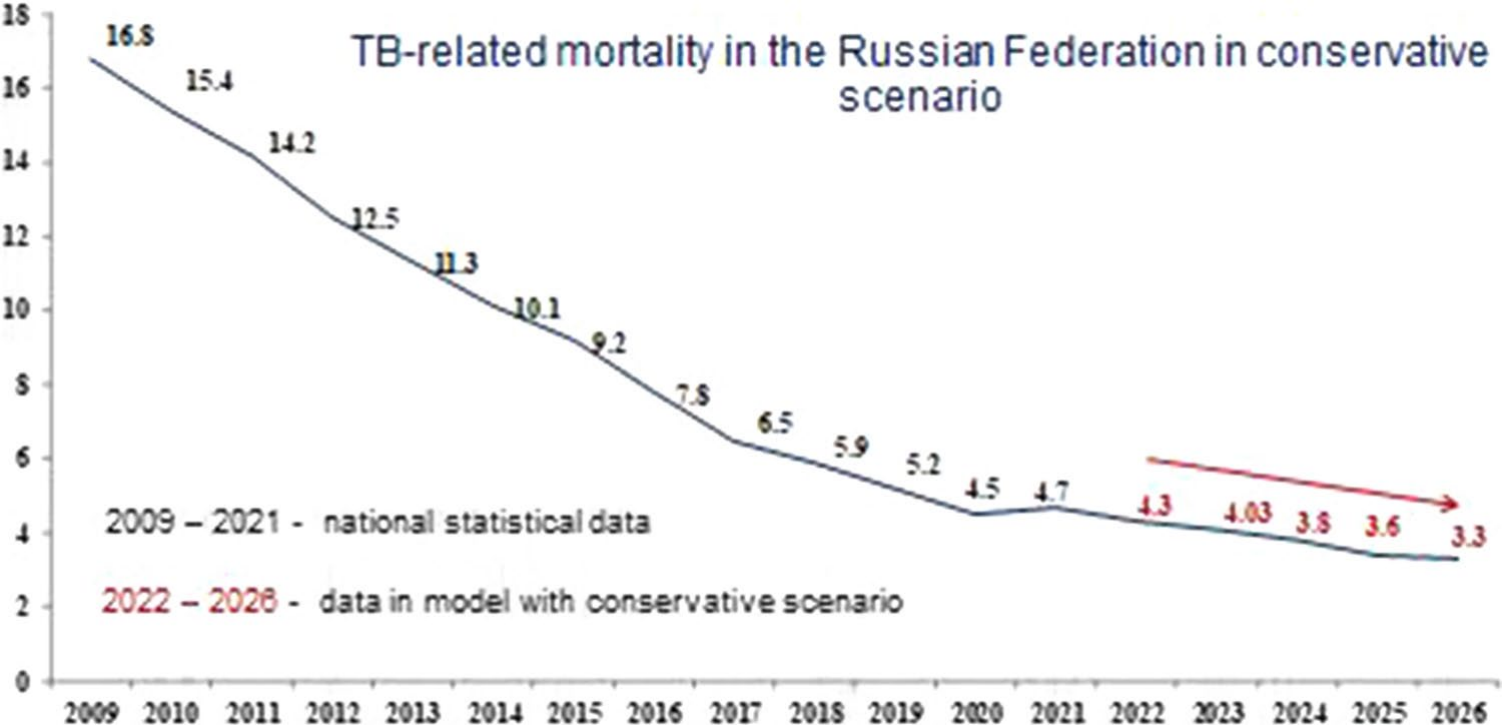
Forecast of TB-related mortality in the Russian Federation with the conservative scenario

**Fig. 9:**
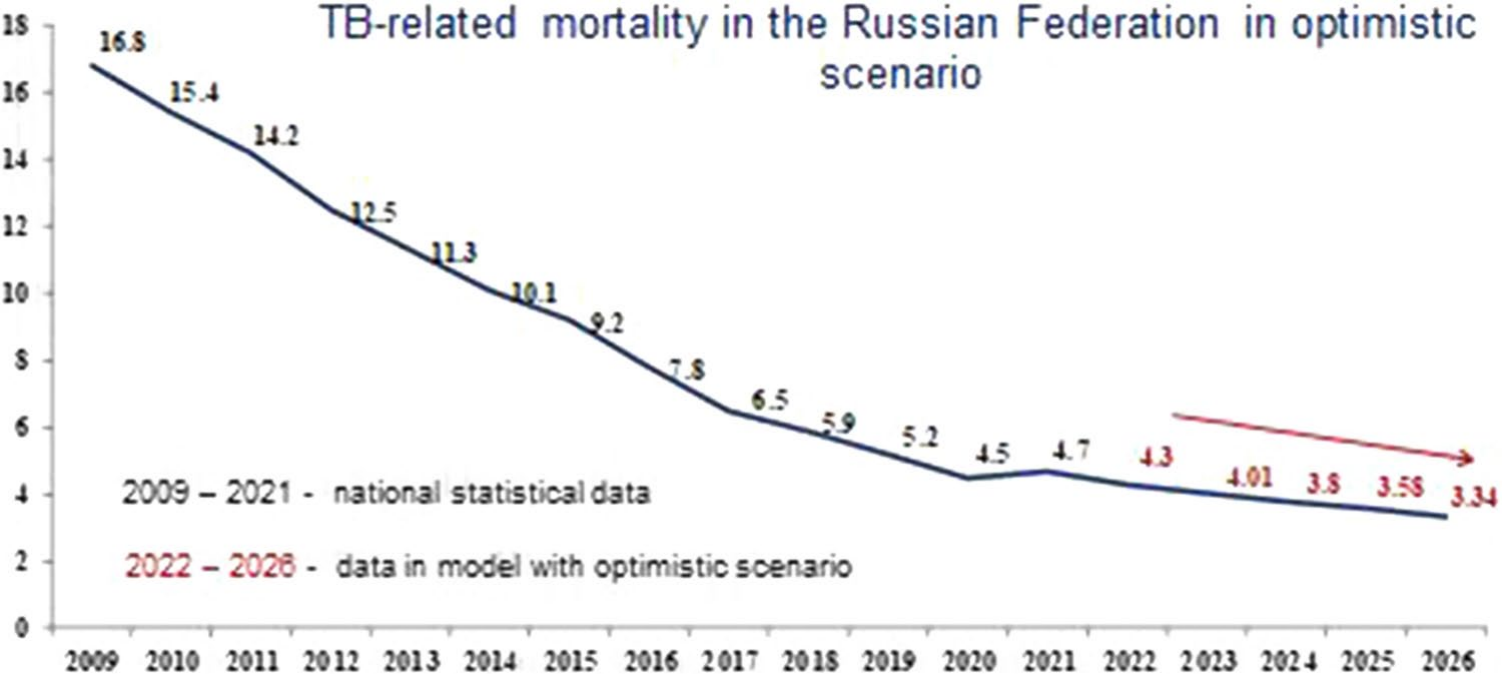
Forecast of TB-related mortality in the Russian Federation with the optimistic scenario

Figures 7, 8 and 9 show the modeling of TB-related mortality rates, taking into account a different scenario for the SE population coverage.

The data presented in Figs. 7, 8 and 9, as well as in the previous versions, various scenarios for the SE population coverage also show a steady decrease in mortality rates in the next 5 years.

We have proved that with the basic scenario for the dynamics in SE coverage, the forecast for mortality in the period from 2022 to 2026 is characterized by a confident downward trend with a decline rate in the range of 5.4–6.4% and a mean decrease rate of 6.0% per year (Fig. 7). Nevertheless, using a conservative scenario for the dynamics of SE coverage, the mortality will continue to decrease with a decrease rate in the range of 5.4–6.7% and a mean decrease rate of 6.0% per year (Fig. 8). With the optimistic scenario for the dynamics of SE coverage, TB-related mortality will also continue to decrease with a decrease rate in the range of 5.4–6.7% and a mean decrease rate of 6.0% per year (Fig. 9).

## Discussion

It is currently clear that the COVID-19 pandemic adversely affected the fight against tuberculosis in all countries worldwide, with serious consequences of the spread of infection and a predicted increase in the number of new cases of the disease in the coming years [2, 10, 21].

In the Russian Federation, epidemiology data of TB was successfully monitored for many years since the beginning of the 1920s, which allows analyzing the features of the epidemiological process over decades, taking into account the impact of various factors [22–24].

Since 2018, WHO has been paying special attention to the problems of detecting and treating tuberculosis in children [3]. In 2020, 226,000 children under the age of 15 years died from TB. Modeling has shown that 80% of TB-related deaths occur in children under 5 years of age, and 96% of children who die from tuberculosis do not receive treatment [25].

In the Russian Federation, immunologic tests are used for TB screening in children: the Mantoux test with 2 TU from the age of 1 year and the test with the recombinant tuberculosis allergen (Diaskintest^®^) from the age of 8, which is used together with a chest X-ray from the age of 15 [26]. In the adult population, depending on the epidemiological situation, TB screening using chest X-ray is carried out once a year or once every 2 years [23, 24].

According to official statistics, TB screening in the Russian Federation has improved over the past 10 years starting from 2009, and the population coverage has increased from 62.5% to 73.7% of the population. In 2020, screening decreased by 7.0–66.7% during the COVID-19 pandemic, following the introduction of maximum restrictive measures with the suspension of elective medical care.

**Fig. 10:**
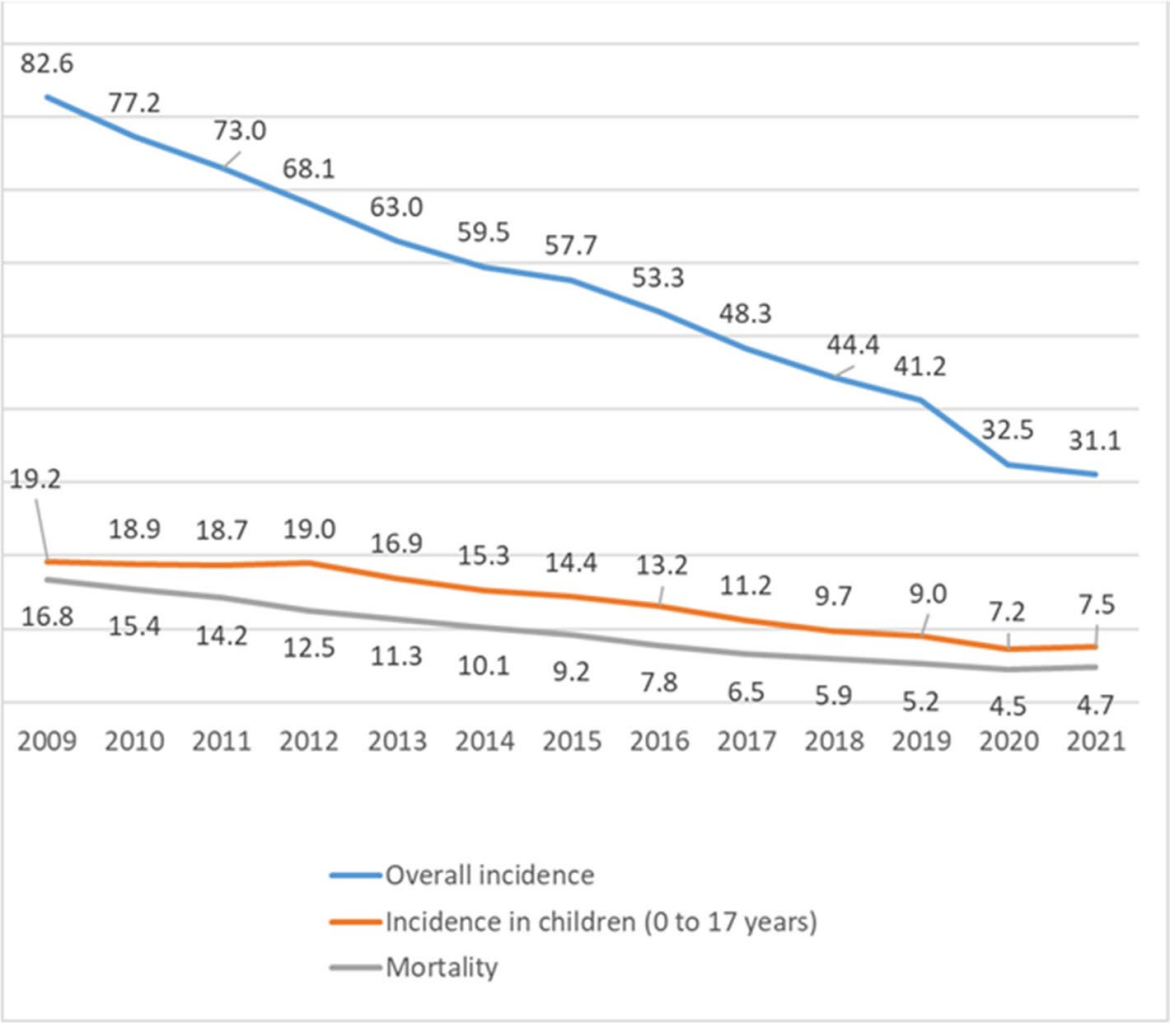
Dynamics of the main epidemiological indicators for tuberculosis in the Russian Federation from 2009 to 2021 per 100,000 population

The measures in the fight against tuberculosis, taken by the Russian Federation in recent years, were most effective and made it possible to systematically reduce the incidence from 82.6 to 31.1 per 100 thousand population from 2009 to 2021 (Fig. 10).

According to the official statistics, a decrease in the overall incidence of tuberculosis in adults and children was observed in the Russian Federation during the COVID-19 pandemic from 2019 to 2021 (Fig. 2). However, these figures cannot be called absolutely reliable, since due to restrictive measures, part of the population was not screened and TB cases could remain undetected.

It should be noted that the incidence of TB in children aged 0–17 years from 2009 to 2020 in the Russian Federation showed a strong downward trend (from 19.2 to 7.2 per 100 thousand population, respectively). However, in 2021, a slight increase in this indicator was noted, which may indirectly indicate an increase in a core of adult smear-positive TB patients.

The TB-related mortality rate in the Russian Federation has also been steadily decreasing from 2009 to 2020 (from 16.8 to 4.5 per 100,000 population, respectively). At the same time, in 2021, the country noted a slight increase in the TB-related mortality rate to 4.7 per 100,000 population, which is not critical, but requires attention.

It should be noted, that in the end of 1980s the TB screening examinations was 75%, which allowed achieving the most favorable incidence of tuberculosis by the end of the 1990s of 34.0 per 100 thousand population [24].

It can be assumed that with a low population coverage with screening examinations, the incidence rates will not be objective due to the lack of information about abnormal changes in the lungs in the unexamined population.

TB screening was well established before the pandemic COVID-19 and have provided decrease in the population coverage in 2020 TB morbidity and mortality drop in the Russian Federation, as can be seen from the 2021 epidemiological data [21, 27].

The possibility of apply the most significant epidemiological indicators of the last decades in the analysis enabled simulation of the situation with tuberculosis in the coming years, which can be a key moment in preventing an increase in disease-related mortality and identifying both adults and children with severe tuberculosis.

Until recently, chest X-ray was considered the most effective method for TB screening [28–30]. However, the emergence of new computer technologies that allow more productive analysis of a large amount of data with greater diagnostic efficiency has challenged the established opinion regarding the X-ray method [31].

At the same time, some studies have shown the effectiveness of chest X-ray only after preliminary sputum smear microscopy, which increases the economic benefits of screening by 19% and the effectiveness of activities, and prevents additional economic losses by 37% [32].

The use of a comprehensive examination with the inclusion of digital radiography and immunological tests in symptomatic patients in a metropolis also turned out to be more effective than a total screening of the population using only an X-ray examination in people without indications for examination [33, 34].

In this study, we analyzed the main epidemiological indicators, taking into account the different population coverage with TB screening examinations in the conditions of the new COVID-19 pandemic and after its end. Predictive models were constructed based on a decrease in the SE population coverage and the impact of external factors (conservative model) or an increase in the coverage when mobilizing existing service resources (optimistic model). A prognostic model of epidemiological situation development using one predictor showed that a change in the SE coverage allows maintaining a downward trend in the incidence of tuberculosis under any scenario. The study shows that if any of the proposed approaches and scenarios are maintained, the incidence of tuberculosis and mortality from the disease will systematically decrease by 2026.

It has been proven that the improvement of the epidemiological situation will not change with a decrease in the scope of screening examinations while maintaining the SE coverage at 65% over a 5-year period, but subsequently, with the accumulation of a reservoir of infection, it can lead to an increase in new cases of TB in patients over the long term.

It should be taken into account that tuberculosis is an infection that responds to external factors with a change in the epidemiological situation after more than 5 years [35]. The economic and social crisis of 1991 in Russia, with a decrease in TB screening to less than 50% of the population, has led to a threefold increase in the incidence of tuberculosis in 2000 to 90.7 per 100,000 population [19, 36], which has also affected the growth of morbidity in the pediatric population in the country [24].

When evaluating the contribution of the predictor to the model of TB incidence in children aged 0–17 years, a direct relationship was established between the population coverage with screening examinations and the incidence of tuberculosis in children (the incidence in children increases with an increase in coverage and, conversely, decreases with a decrease in coverage). The resulting dependence is natural, since screening for tuberculosis infection in children includes a Mantoux test with 2 TU from the first year of life to 8 years, then a test with Diaskintest up to 14 years, and an immunological test together with a chest X-ray from 15 to 17 years [37, 38]. This approach enables the most effective detection of latent and active tuberculosis in children in a country with a high TB burden [16, 26].

The study analyzed epidemiological indicators for a period of 12 years and assessed the impact of only one factor—the population coverage with TB screening examinations. Based on the data obtained, it can be argued that to improve the accuracy of modeling and forecasting the epidemiological situation for tuberculosis, it is necessary to search for additional, more informative indicators that would allow us to more reliably influence the change in epidemiological indicators.

Perhaps, in the coming years, the use of alternative methods of machine learning and artificial intelligence will enable the analysis of computed tomography data, which, given the large number of examinations conducted during the COVID-19 pandemic, may be the most effective [39].

## Conclusion

An analysis of epidemiological data on tuberculosis infection before the start of the COVID-19 pandemic showed a decrease in the incidence and mortality rates after 2020 in the Russian Federation as a whole. At the same time, according to the World Health Organization Global Tuberculosis Report 2021, there is an increase in mortality rates from tuberculosis in the world.

When constructing a predictive model for the development of the epidemiological situation for tuberculosis in the Russian Federation on the basis of a single predictor—the population coverage with TB screening examinations, it was found that to maintain the trend toward a decrease in TB incidence and mortality, the population coverage with screening examinations using chest X-ray is not sufficient. However, to maintain a positive trend in reducing TB incidence and mortality, population coverage with screening should be at least 60%, while maintaining screening in children using immunodiagnostic tests at a level of at least 90%. It should be noted that the possibility of revising the screening conditions with use of a more comprehensive examination in the risk groups and symptomatic patients, including microscopy, molecular genetic methods and computed tomography, can optimize costs and improve the diagnosis of tuberculosis with a more effective and targeted focus on detection of the disease.

## Data Availability

All data generated or analyzed during this study are included in this published article.

## Abbreviations

TB: Tuberculosis
DOTS: Directly observed treatment, short-course
COVID-19: New coronavirus infection
HIV: Human immunodeficiency viruses
WHO: World Health Organization
AR: Autoregression
SE: Screening examination
